# Association between Internet Use Behavior and Palpitation among Adolescents: A Cross-Sectional Study of Middle School Children from Northwest Romania

**DOI:** 10.3390/ijerph17124278

**Published:** 2020-06-15

**Authors:** Cecilia Lazea, Alexandra Popa, Cristina Varga

**Affiliations:** 1 Department Pediatrics I, Emergency Pediatric Hospital, University of Medicine and Pharmacy ”Iuliu Hatieganu”, 400370 Cluj-Napoca, Romania; alexindra.popa@gmail.com; 2 Centre Hospitalier Universitaire (CHU), 67299 Strasbourg, France; cristina.pihuleac@gmail.com

**Keywords:** internet, adolescents, cardiac symptoms

## Abstract

Purpose: The use of the internet is a tool and media literacy has become an essential skill among adolescents. Related to this behavior, some adolescents evoke cardiovascular effects. The purpose of this study was to explore a possible correlation between internet use behavior and occurrence of palpitations and related symptoms among a representative cohort of adolescents from the north-west region of Romania. Method: The study included students of seven middle schools from Northwest Romania. Participants completed an anonymous questionnaire consisting of 18 questions about internet use. Results: In total, 1147 students responded to the study. Mean duration of daily internet usage was 2.57 h during school time and 3.57 h during the holidays. A total of 77% of adolescents had more than one symptom related to internet use, and 11% of them reported palpitations and related symptoms. We found an independent relation between palpitation and urban background, palpitations and the internet usage time interval 20:00–24:00, and palpitations and tobacco smoking. Strong heartbeats were independently associated with the time interval 12:00–16:00, tobacco smoking, and energy drink consumption. Conclusion: In our cohort, the most important factors associated with the occurrence of palpitations and related symptoms were the timeframe of internet usage and smoking.

## 1. Introduction

The Internet is both a worldwide tool for information dissemination and a medium of interaction between individuals using their computers, regardless of geographic location. The Internet has completely reshaped our lives, from how we communicate to how we consume news, shop, navigate, and entertain ourselves. Unfortunately, the Internet also has a dark side; excessive internet use can lead to addiction, emotional disruption, and physical health issues [1,2].

According to Internet World Stats and European Commission, as of June 2019, the internet penetration rate in Europe was 87.2%, with more than 73% of Romanian households using a fixed broadband service or subscribing to mobile services [3,4]. Among those customers, 88% were adolescents who spent more than 8 h per day in front of screens (TV, video games, and internet). Adolescence is a transitional stage characterized by turbulent cognitive, social, and somatic development. Since teenagers can be socially and emotionally fragile, excessive internet use at this age can impair mental health and have harmful effects, including internet addiction [1,2,5]. Various studies have revealed changes in the brains of individuals with behavioral addiction—disruption in the functional connection between regions located in the frontal, occipital, and parietal lobes in internet addiction patients, or diminished error-related anterior cingulated cortex activity [5,6,7]. A study by Cheng et al. that included seven world regions and 31 nations showed that extensive internet use is a serious and widespread problem, especially in economically developed countries [8]. There are two hypotheses related to extensive internet use: (1) the accessibility hypothesis, which claims that wider internet availability leads to a greater engagement in online activities, and (2) the quality of life hypothesis, which predicts that, in countries where quality of life is poor, the incidence of internet addiction is higher, most likely due to individuals using virtual reality to escape from everyday problems [8,9].

Extensive internet use may be a non-substance form of addiction and is highly prevalent among adolescents, who are in a critical period for developing a mature amygdala–prefrontal cortex circuit and building up emotional regulatory ability [10,11]. Extensive internet use may lead to psychosocial problems, somatic diseases, and worsened school performance [12]. However, several studies from other European countries (as well as Romania) have analyzed psychosocial issues related to excessive internet usage and internet addiction: suicidal behaviors (suicidal ideation and suicide attempts); depression; anxiety; conduct problems and hyperactivity/inattention; emotional, self-control, and behavioral issues; aggression; and sleeping problems [13,14,15,16]. Little attention has been paid to the physical health effects of internet use among adolescents, although a few studies have investigated the effects of internet use on sleep duration, eating disorders, eye problems, headaches, tiredness, and increased risk for unnatural death, but there are no studies that have evaluated the possible cardiovascular effects of extensive internet use [16,17,18,19,20,21]. To the best of our knowledge, no large-scale studies on effects of the excessive internet use among Romanian teenagers have been conducted.

The objective of this study was to evaluate the association between palpitations and related symptoms and internet use in a sample of Romanian adolescents from urban areas.

## 2. Materials and Methods

A descriptive cross-sectional study was conducted to examine the association between palpitations and related symptoms, and internet use in adolescents from urban areas. This study attempted to provide data to promote a healthy lifestyle at this age group.

### 2.1. Setting and Sample

The study’s participants included 1300 middle school students between the ages of 13 and 15 from seven schools in two cities in northwest Romania. The schools were socioeconomically comparable. Of these, 153 students that had missing data were excluded.

### 2.2. Measurement

Participants filled out a self-administered questionnaire which asked about socio-demographic factors (age, gender, and residential area); internet usage duration during the school year and vacations; sleep duration; height and weight; tobacco smoking; coffee, caffeinated soft drink, and energy drink consumption during internet usage; and somatic symptoms. The cardiovascular symptoms assessed were palpitations and related symptoms (tachycardia and strong heartbeats). Palpitations were explained to the participants as a sensation that the heart has skipped a beat or added an extra beat, or a fluttering and racing heart. Tachycardia was explained as abnormally rapid regular heartbeats. Strong heartbeats were explained as stronger or more vigorous heartbeats than usual.

Several aspects of internet behavior were studied: frequency, duration, timing, and purpose of internet use. Internet usage duration was quantified using the time interval 08:00 a.m. to 12:00 p.m.; 12:00 p.m. to 04:00 p.m.; 04:00 p.m. to 08:00 p.m.; 08:00 p.m. to 12.00 a.m., and 12:00 a.m. to 08:00 a.m. Internet usage and sleep duration were analyzed during the school year and vacations.

### 2.3. Data Collection and Ethical Considerations

The anonymous self-administered questionnaire was completed in a 20-min session in students’ classrooms. Participants were supervised by teachers, who provided necessary explanations, especially for cardiovascular terms, in order to minimize potential information bias.

The students and their supervisors were informed about the purpose of the study and the questionnaire structure. Written informed consent was obtained from the family of each student. The nature of the study was fully explained to teachers at the participating schools, and their permission to conduct this study was verbally obtained. The research was approved by the County School Inspectorates, and relevant permissions were obtained from each school.

### 2.4. Statistical Analysis

Statistical analyses were performed using MedCalc version 19.0.3 (MedCalc Software, Ostend, Belgium). Qualitative variables were characterized by frequency and percentage. Comparisons between groups were made using chi-squared tests, and multivariate analysis was performed with binary logistic regression. Statistical significance was established by calculating the *p*-value, with a statistical significance cutoff of 0.05.

## 3. Results

### 3.1. Demographics, Duration of Internet Use, Internet Activities, and Sleep Duration

A total of 1147 eligible participants (46.8% female, 53.2% male; mean age = 13.86 ± 0.76 years, range 12–15) responded to the present study. The response rate was 88.2%. Data analysis was targeted at school periods as well as school vacations. The socio-demographic data are presented in Table 1.

The mean duration of daily internet use was 2.57 h during school periods and 3.57 h during vacations. The most common internet activities among participants were online communication using social networking sites (94.9%) and playing online games (62.16%). Of all adolescents surveyed, 57% admitted consuming caffeinated beverages and smoking during internet use. A high number of adolescents reported experiencing various symptoms during internet use: dizziness (14.03%), fainting (2.87%), nervousness (16.21%), sleepiness (23.36%), insomnia (10.46%), lack of concentration (11.85%), sadness (11.94%), boredom (4.09%), and headache (14.99%). Moreover, 11% of participants reported experiencing palpitations, tachycardia, and strong heartbeats, and 77% had more than one symptom.

Participants’ internet usage duration and sleep duration data are presented in Table 2.

### 3.2. Internet Use and Cardiovascular Symptoms

The general characteristics of patients with palpitations and related symptoms are summarized in Table 3.

No significant correlation was found between palpitations, tachycardia, or strong heartbeats and age and sex.

In order to find out which variables were independently associated with the presence of palpitations and related symptoms, we used logistic regression (Table 4, Table 5 and Table 6). The following parameters were introduced: residential area, smoking, energy drink intake, caffeinated soft drink intake, and time interval of internet use during the school period and holidays. A statistically significant correlation was noted between heart palpitations and residential area when multiple logistic binary regression was used to determine the independent association between palpitations and residence in an urban area, which was identified as the independent variable related to the presence of heart palpitations (*p* = 0.04).

Participants’ activities during internet use and time interval of internet use are presented in Table 7.

There was no difference between school and vacation periods in the occurrence of heart palpitations at night (8:00 p.m. to 12:00 a.m. and 12:00 a.m. to 8:00 a.m.; *p* = 0.01), nor between tachycardia and strong heartbeats in the morning (8:00 a.m. to 12:00 p.m.; *p* = 0.04; *p* = 0.02 for school period and *p* = 0.0004 for vacations). In vacation periods, tachycardia occurred most often between 12:00 a.m. and 8:00 a.m. (*p* = 0.007) and strong heartbeats between 12:00 p.m. and 4:00 p.m. (*p* = 0.01). Activities carried out online were not correlated with the appearance of palpitations and related symptoms. A multivariate analysis of internet activities during vacations demonstrated an independent relation between heart palpitations and internet use during the time interval of 8:00 p.m. to 12:00 a.m. (*p* = 0.05). Tachycardia was independently associated with the time interval of 12:00 a.m. to 8:00 a.m. (*p* = 0.08), while strong heartbeats were independently associated with the time interval 12:00 p.m. to 4:00 p.m. (*p* = 0.04).

All students who experienced these manifestations were tobacco smokers and regularly consumed carbonated drinks that enhance cardiac activity (Table 8).

The occurrence of heart palpitations and strong heartbeats among tobacco smokers and consumers of energy drinks, as well as consumers of caffeinated soft drinks, was statistically significant, and all these symptoms mentioned in the study were present. The number of coffee cups taken had no apparent relation to the occurrence of palpitations and related symptoms. Our multivariate analysis demonstrated an independent relation only between palpitations and tobacco smoking (*p* = 0.03), while strong heartbeats were independently associated with tobacco smoking (*p* = 0.01) and energy drink consumption (*p* = 0.05).

## 4. Discussion

In this study, we aimed to establish a possible correlation between internet use, the associated consumption of substances with cardiovascular stimulant potential, and the occurrence of palpitations and related symptoms. Excessive computer use or internet addiction has a very high prevalence rate among adolescents and children. The present study focused on teenagers between 13 and 15 years old.

We found that urban environment was an independent factor for the appearance of palpitations. Stress is known to be a serious health risk in modern everyday life, and the urban environment involves a combination of specific factors that contribute to both mental and physical stress, whether directly or indirectly. On the other hand, nature—the defining feature of conserved rural environments—can promote stress recovery and general well-being [22]. While we have easy access to the Internet, excessive time spent on computers may result in physical damage, including musculoskeletal injuries, vision problems, nutritional deficiencies, and sleep deprivation, as well as negative psychosocial effects, such as depression, aggressive behavior, problems in interpersonal relationships, anxiety, hyperactivity, or inattention [13,14,15,16,17,18,19,20]. The consequences of unrestricted internet use on physical health have been less investigated than its effects on psychological health.

Our study showed that the majority of adolescents use the Internet daily for varying lengths of time depending on their school schedules: 2.57 h during school periods and 3.57 h during vacations. It is worth mentioning that 55% of adolescents who used the internet for more than 3 h per day during vacations described this activity as a “favorite” activity, while during school periods only 24% of children spent more than 3 h on the Internet daily at the expense of school-related activities (*p* = 0.0033). In comparing average duration of internet usage, we found no statistically significant differences between the groups who used the internet for 3 or 4 h per day during the two time periods.

During school periods, the preferred time interval for internet use was immediately after class (4:00 p.m. to 8:00 p.m.), while during vacations, internet use was somewhat balanced over the course of the day. However, we noticed a preference for online activities in the afternoon and late evening—even after midnight—with a subsequent alteration of adolescents’ sleep schedules. Mean sleep duration in our group was 7.92 h during school periods and 8.61 h during vacations, with no statistically significant difference, suggesting that many adolescents sleep less than recommended, 8 to 10 h/day [23]. Previous research has noted the association between extensive internet usage and shorter sleep duration, poor sleep quality, altered circadian rhythms, and other somatic and psychological symptoms [17,24,25,26].

In our study, independent correlations between palpitations and related symptoms were established as the following: during the time interval 8:00 p.m. to 12:00 a.m. for tachycardia, during the time interval 12:00 p.m. to 16:00 p.m. for strong heartbeats, and during the time interval 08:00 p.m. to 12:00 a.m. for palpitations (OR = 3.38; OR = 3.14, and OR = 3.62, respectively). Even though we found strong statistical evidence that some time intervals of internet usage were independent risk factors for these symptoms, we did not identify any certain causality for those effects. We thus assume that this was due to a third factor. In light of the findings of other studies, we cannot rule out the possibility that shorter sleep duration could be the result of symptoms that occur after extensive internet use, since the excessive use of electronic media leads to less sleep [17,26]. This observation is consistent with studies that have demonstrated reduced autonomic flexibility, impaired control of emotions, and anxiety in problematic internet users, similar to substance addictions (where autonomic functioning is also impaired), and that excessive internet use affects the sympathetic nervous system and dopaminergic pathways associated with increased systolic blood pressure, increased heart rate, and compromised immune function [27,28,29].

Overall, sleep duration in Romanian adolescents is not significantly higher than in other populations: 7.9 h in Finland, 7.85 h in France, 7.6 h in Denmark, and 7.55 h in Hong Kong [17]. Parental monitoring is very important in addressing this issue. When parents leave adolescents in their bedroom thinking that they will fall asleep, children in fact often continue to use electronic devices, very often not for completing their academic work [24]. Time is an important resource, and it is more beneficial to spend time on study or physical activity than on unnecessary internet activities.

We did not find any statistically significant association between type of online activity and occurrence of palpitations and related symptoms. However, the most prevalent internet activities among participants in our study were online communication using social networking sites, especially Facebook (85%) and online gaming (74%), which are risk factors associated with excessive internet use [30]. Those results are consistent with Tsitsika et al.’s study, which showed that 82% of European and American adolescents had a social networking profile and more than half used social networking sites daily [15]. These activities are considered a normal adolescent practice and form an integral part of teenage social life, although they have various negative physical and psychological effects as well as privacy implications, such as isolation and exposing children to online predators [25,31]. Studies have claimed that adolescents mainly use social networks to keep in touch with other individuals and use them much less often for academic purposes [27].

In a recent study, Wassef et al. found that energy drink consumption was an additional causative factor for tachycardia [32]. Previous studies have demonstrated increased systolic blood pressure, altered electrolytes, and resulted repolarization abnormalities as cardiovascular responses to energy drinks in healthy populations [33,34]. The consumption of carbonated sugar-sweetened beverages, even if it does not have immediate effects, is also linked to chronic health problems, including cardiovascular diseases, obesity, type 2 diabetes, non-alcoholic fatty liver disease, and gout [34,35]. With regards to the consumption of beverages with cardio-stimulating effects, all adolescents with palpitations and related symptoms in our study consumed coffee, caffeinated soft drinks, or energy drinks. The association between caffeinated soft drink consumption and the presence of these symptoms mentioned in the study was statistically significant, but cannot be considered an autonomous risk factor. The energy drink intake, more than two cans per day was frequent associated with palpitations (OR = 5.56) and strong heartbeats (OR = 2.61).

An independent correlation was established only between strong heartbeats and consumption of energy drinks. Although all adolescents who exhibited cardiovascular manifestations were coffee users, we found no correlation between the amount of coffee consumed and the onset of symptoms. We therefore deduce that palpitations and related symptoms may be triggered not only by the amount of caffeine consumed but by other ingredients in energy drinks, an observation which is corroborated by other studies [36].

As over 50 years have passed since the harmful health effects of tobacco use were first studied, it is already known that tobacco use is a leading cause of cardiovascular disease morbidity and mortality [37,38]. Here, as in other studies, we obtained a statistically significant association between smoking and palpitations (OR = 3.41); however, is important to note the age at which adolescents start smoking, as the risk of developing nicotine dependence increases in inverse proportion to age of onset [39].

Our study has several limitations. First, it is a cross-sectional study and therefore cannot be used to interpret cause–effect relationships. Since the data were self-reported, stigmatizing behaviors could be unreported or minimized, and healthy behaviors could be over-reported. In order to minimize reporting bias, students were asked to complete the questionnaires in class anonymously. Second, in order to keep the questionnaire to an acceptable length, we did not collect information about parental behavior. However, parental behavior may play an important role as a model for adolescents’ behavior. In addition, we did not record data about the commercial names of energy drinks, which creates a bias in our results due to the differences in caffeine content across various brands of energy drinks. Finally, the study included only seven schools in an urban region of Romania, which may limit the generalizability of the results.

## 5. Conclusions

Our study presents a preliminary association between extensive internet use and palpitations in Romanian adolescents. Higher symptom load was associated with short sleep duration, high-frequency computer use, energy drink consumption, and tobacco smoking. Further studies are needed to corroborate these findings and develop strategies to moderate internet use in children and adolescents.

## Figures and Tables

**Table 1:** Socio-demographic data.

Parameter		Subjects (*n*, *%*)
Age	12 years	9 (0.75%)
	13 years	383 (33.4%)
	14 years	559 (48.7%)
	15 years	196 (18.7%)
Sex	Females	537 (53.18%)
	Males	610 (56.82%)
Residential area	Urban	1053 (91.8%)
	Rural	94 (8.2%)
School enrollment	7th grade	592 (56.54%)
	8th grade	455 (43.46%)

**Table 2:** Internet usage duration and sleep duration data.

Parameters		School Period		School Holiday		*p*-Value
		No.	%	No.	%	
**Frequency of internet connection**	Daily	791	68.96	911	79.42	0.02 *
	Every 2–3 days	266	23.19	139	12.11	0.0001 *
	Weekly	68	5.92	38	3.31	0.04 *
	Every 2 weeks or rarer	22	1.91	59	5.14	0.0005 *
**Daily internet connection time**	0–30 min	126	10.98	46	4.01	0.0001 *
	1 h	241	21.01	115	10.02	0.0001 *
	2 h	218	19.00	138	12.03	0.0007 *
	3 h	264	23.01	218	19.00	0.056
	4 h	149	12.99	195	17.00	0.207
	5 h	69	6.01	160	13.94	0.0001 *
	>5 h	80	6.97	275	23.97	0.0001 *
**The period of the day of internet connection**	8:00 a.m. to 12:00 p.m.	23	2.00	138	12.03	0.0001 *
	12:00 p.m. to 4:00 p.m.	161	14.03	229	19.96	0.014 *
	4:00 p.m. to 8:00 p.m.	642	55.97	298	25.98	0.0001 *
	8:00 p.m. to 12:00 p.m.	275	23.97	310	27.02	0.19
	12:00 a.m. to 8:00 a.m.	46	4.01	172	14.99	0.0001 *
**Sleep duration**	<8 h	385	33.56	304	26.51	0.67
	>8 h	762	66.43	843	73.49	0.12

* Significance level of *p* < 0.05.

**Table 3:** General characteristics of subjects with palpitations and related symptoms.

Demographic Characteristics		Symptoms					
		Palpitations (*n*, %)	*p*	Tachycardia (*n*, %)	*p*	Strong Heartbeats (*n*, %)	*p*
		15		46		24	
**Age (years)**	13 years	3 (20%)	0.3	12 (26.1%)	0.4	7 (29.2%)	0.6
	14 years	10 (66.7%)		27 (58.7%)		14 (58.3%)	
	15 years	2 (13.3%)		7 (15.2%)		3 (12.5%)	
**Gender**	Male	10 (66.7%)	0.4	28 (60.9%)	0.3	13 (54.2%)	0.6
	Female	5 (33.3%)		18 (39.1%)		11 (45.8%)	
**Environment**	Rural	4 (26.7%)	0.02 *	7 (15.2%)	0.1	3 (12.5%)	0.6
	Urban	11 (73.3%)		39 (84.8%)		21 (87.5%)	

* Significance level of *p* < 0.05.

**Table 4:** Association between palpitations and other variables.

Palpitations		B	*p*	OR	95% CI for OR
**Residential area**	Urban	−1.59	0.01	0.20	[0.05,0.71]
**Smoking (cigarettes/day)**	5–10	-	0.14	-	-
	10–20	1.22	0.06	3.41	[0.91,12.72]
	>20	1.06	0.32	2.91	[0.34,24.71]
**Energy drink intake (cans/day)**	1	-	0.10	-	-
	2	1.13	0.10	3.12	[0.79,12.19]
	>2	1.71	0.03	5.56	[1.09,28.42]
**Time interval of internet use during school period**	8:00 p.m. to 12:00 a.m	0.52	0.37	1.69	[0.52,5.45]
	12:00 a.m. to 8:00 a.m.	0.45	0.57	1.57	[0.32,7.61]
**Time interval of internet use during holidays**	8:00 p.m. to 12:00 a.m.	1.28	0.04	3.62	[1.04,12.61]
	12:00 a.m. to 8:00 a.m.	0.58	0.34	1.78	[0.54,5.90]

**Table 5:** Association between tachycardia and other variables.

Tachycardia		B	*p*	OR	95% CI for OR
**Caffeinated soft drink intake** **(liters/day)**	<0.5		0.09		
	0.5–1	1.49	0.15	4.44	[0.55,35.54]
	1–2	1.11	0.34	3.04	[0.30,30.44]
	>2	2.67	0.01	14.52	[1.58,132.86]
**Time interval of internet use during school period**	8:00 p.m. to 12:00 a.m.	0.47	0.47	1.61	[0.43,6.03]
	12:00 a.m. to 8:00 a.m.	0.83	0.13	2.30	[0.77,6.82]
**Time interval of internet use during holidays**	8:00 a.m. to 12:00 p.m.	1.21	0.00	3.38	[1.76,6.46]

**Table 6:** Association between strong heartbeats and other variables.

Strong Heartbeats		B	*p*	OR	95% CI for OR
**Caffeinated soft drink intake (liters/day)**	<0.5		0.00		-
	0.5–1	−0.20	0.86	0.81	[0.07,8.44]
	1–2	−0.60	0.66	0.54	[0.03,8.26]
	>2	2.41	0.04	11.19	[1.11,112.15]
**Energy drink intake (cans/L)**	1	-	0.35		-
	2	0.50	0.32	1.64	[0.61,4.43]
	>2	0.95	0.17	2.61	[0.65,10.39]
**Time interval of internet use during school period**	8:00 a.m. to 12:00 p.m.	0.53	0.50	1.70	[0.35,8.28]
**Time interval of internet use during holidays**	8:00 a.m. to 12:00 p.m.	0.67	0.22	1.97	[0.66,5.85]
	12:00 p.m. to 16:00 a.m.	1.14	0.03	3.14	[1.11,8.90]
	8:00 p.m. to 12:00 a.m.	0.76	0.11	2.14	[0.83,5.52]

**Table 7:** Activities during internet use, time interval, and their correlation with palpitations and related symptoms.

		Palpitations (*n*, %)	*p*	Tachycardia (*n*, %)	*p*	Strong Heartbeats (*n*, %)	*p*
**Activities on the Internet**	Gaming	10 (66.7%)	0.9	34 (73.9%)	0.1	19 (79.2%)	0.1
	Social media	13 (86.7%)	0.9	38 (82.6%)	1	22 (91.7%)	0.3
	Documenting	9 (60.0%)	0.9	27 (58.7%)	0.8	16 (66.7%)	0.3
	E-mail	4 (26.7%)	0.5	18 (39.1%)	0.4	9 (37.5%)	0.6
	Blogging	2 (13.3%)	0.8	8 (17.4%)	0.08	4 (16.7%)	0.2
**Time interval of internet use during school period**	8:00 a.m. to 12:00 p.m	2 (13.3%)	0.09	4 (8.7%)	0.04 *	3 (12.5%)	0.02 *
	12:00 p.m. to 4:00 p.m.	4 (26.7%)	0.4	10 (21.7%)	0.4	6 (25.0%)	0.4
	4:00 p.m. to 8:00 p.m.	7 (46.7%)	0.2	31 (67.4%)	0.8	17 (70.8%)	0.7
	8:00 p.m. to 12:00 a.m.	9 (60.0%)	0.01 *	15 (33.3%)	0.4	10 (41.7%)	0.1
	12:00 a.m. to 8:00 a.m.	3 (20.0%)	0.01 *	6 (13.0%)	0.007 *	3 (12.5%)	0.1
**Time interval of internet use during holidays**	8:00 a.m. to 12:00 p.m.	5 (33.3%)	0.1	19 (41.3%)	0.0004 *	10 (41.7%)	0.004 *
	12:00 p.m. to 4:00 p.m.	5 (33.3%)	1	17 (37.0%)	0.3	13 (54.2%)	0.01 *
	4:00 p.m. to 8:00 p.m.	6 (40.0%)	1	23 (50.0%)	0.1	11 (45.8%)	0.6
	8:00 p.m. to 12:00 a.m.	11 (73.3%)	0.01 *	18 (39.1%)	0.9	15 (62.5%)	0.04 *
	12:00 a.m. to 8:00 a.m.	8 (53.3%)	0.01	15 (32.6%)	0.1	9 (37.5%)	0.1

* Significance level of *p* < 0.05.

**Table 8:** Correlation between beverage intake and tobacco smoking during internet use and palpitations and related symptoms.

Activity		Palpitations (*n*, %)	*p*	Tachycardia (*n*, %)	*p*	Strong Heartbeats (*n*, %)	*p*
**Tobacco smoking (cigarettes/day)**	<5	0 (0%)	0.002 *	0 (0%)	0.4	0 (0%)	0.00 *
	5–10	10 (66.7%)		40 (87.0%)		17 (70.8%)	
	10–20	4 (26.7%)		5 (10.9%)		6 (25%)	
	>20	1 (6.7%)		1 (2.2%)		1 (4.2%)	
**Caffeinated soft drinks intake (liters)**	<0.5	7 (47.7%)	0.01 *	28 (60.9%)	0.04 *	12 (54.2%)	0.00 *
	0.5–1	3 (20.0%)		10 (21.7%)		4 (16.7%)	
	1–2	2 (13.3%)		3 (6.5%)		2 (8.3%)	
	>2	3 (20.0%)		5 (10.9%)		5 (20.8%)	
**Coffee intake** **(cups)**	1	10 (66.7%)	0.8	34 (73.9%)	0.1	17 (70.8%)	0.9
	2	4 (26.7%)		8 (17.4%)		6 (25.0%)	
	>2	1 (6.7%)		4 (8.7%)		1 (4.2%)	
**Energy drinks intake (cans)**	1	3 (20.0%)	0.001 *	21 (45.7%)	0.1	9 (37.5%)	0.01 *
	2	8 (53.3%)		19 (41.3%)		10 (41.7%)	
	>2	4 (26.7%)		6 (13.0%)		5 (20.8%)	

* Significance level of *p* < 0.05.

## References

[1] Koyuncu T., Unsal A., Arslantas D. (2014). Assessment of internet addiction and loneliness in secondary and high school students. J. Pak. Med. Assoc..

[2] Weinstein A., Yaacov Y., Manning M., Danon P., Weizman A. (2015). Internet Addiction and Attention Deficit Hyperactivity Disorder Among Schoolchildren. Isr. Med. Assoc. J. IMAJ.

[3] Internet World Stats European Internet and Population Statistics. https://www.internetworldstats.com..

[4] European Union Digital Economy and Society Statistics-Households and Individuals. https://ec.europa.eu.

[5] Yau Y.H.C., Potenza M.N., Mayes L.C., Crowley M.J. (2015). Blunted feedback processing during risk-taking in adolescents with features of problematic Internet use. Addict. Behav..

[6] Wee C.-Y., Zhao Z., Yap P.-T., Wu G., Shi F., Price T., Du Y., Xu J., Zhou Y., Shen D. (2014). Disrupted Brain Functional Network in Internet Addiction Disorder: A Resting-State Functional Magnetic Resonance Imaging Study. PLoS ONE.

[7] Chung T., Sum S.M., Chan M.W. (2019). Adolescent Internet Addiction in Hong Kong: Prevalence, Psychosocial Correlates, and Prevention. J. Adolesc. Health.

[8] Cheng C., Li A.Y.-L. (2014). Internet Addiction Prevalence and Quality of (Real) Life: A Meta-Analysis of 31 Nations Across Seven World Regions. Cyberpsychol. Behav. Soc. Netw..

[9] Tang Z., Zhou S. (2009). Psychological risk factors of the youth with Internet addiction. Chin. J. Clin. Psychol..

[10] Victorin Å., Johnels J.Å., Bob E., Kantzer A., Gillberg C., Fernell E. (2020). Significant gender differences according to the Problematic and Risky Internet Use Screening Scale among 15-year-olds in Sweden. Acta Paediatr..

[11] Cheng H., Liu J. (2020). Alterations in Amygdala Connectivity in Internet Addiction Disorder. Sci. Rep..

[12] Javaeed A., Jeelani R., Gulab S., Ghauri S.K. (2020). Relationship between internet addiction and academic performance of undergraduate medical students of Azad Kashmir. Pak. J. Med Sci..

[13] Kaess M., Durkee T., Brunner R., Carli V., Parzer P., Wasserman C., Sarchiapone M., Hoven C., Apter A., Balazs J. (2014). Pathological Internet use among European adolescents: Psychopathology and self-destructive behaviours. Eur. Child Adolesc. Psychiatry.

[14] Blinka L., Skarupova K., Sevcikova A., Wölfling K., Müller K.W., Dreier M. (2014). Excessive internet use in European adolescents: What determines differences in severity?. Int. J. Public Health.

[15] Tsitsika A.K., Tzavela E.C., Janikian M., Ólafsson K., Iordache A., Schoenmakers T.M., Tzavara C., Richardson C. (2014). Online Social Networking in Adolescence: Patterns of Use in Six European Countries and Links With Psychosocial Functioning. J. Adolesc. Health.

[16] Smahel D., Wright M.F., Černíková M. (2015). The impact of digital media on health: Children’s perspectives. Int. J. Public Health.

[17] Nuutinen T., Roos E., Ray C., Villberg J., Välimaa R., Rasmussen M., Holstein B.E., Godeau E., Beck F., Grippe T. (2014). Computer use, sleep duration and health symptoms: A cross-sectional study of 15-year olds in three countries. Int. J. Public Health.

[18] Alpaslan A.H., Kocak U., Avci K., Taş H.U. (2015). The association between internet addiction and disordered eating attitudes among Turkish high school students. Eat. Weight Disord. Stud. Anorexia Bulim. Obes..

[19] Sampasa-Kanyinga H., Chaput J.-P., Hamilton H.A. (2015). Associations between the use of social networking sites and unhealthy eating behaviours and excess body weight in adolescents. Br. J. Nutr..

[20] Lin P., Kuo S.-Y., Lee P.-H., Sheen T.-C., Chen S.-R. (2014). Effects of Internet Addiction on Heart Rate Variability in School-Aged Children. J. Cardiovasc. Nurs..

[21] Fu K.-W., Chan C.-H., Ip P. (2014). Exploring the relationship between cyberbullying and unnatural child death: An ecological study of twenty-four European countries. BMC Pediatr..

[22] Corazon S.S., Sidenius U., Poulsen D.V., Gramkow M.C., Stigsdotter U. (2019). Psycho-Physiological Stress Recovery in Outdoor Nature-Based Interventions: A Systematic Review of the Past Eight Years of Research. Int. J. Environ. Res. Public Health.

[23] Chaput J.-P., Dutil C., Sampasa-Kanyinga H. (2018). Sleeping hours: What is the ideal number and how does age impact this?. Nat. Sci. Sleep.

[24] Short M.A., Gradisar M., Wright H., Lack L.C., Dohnt H., Carskadon M.A. (2011). Time for Bed: Parent-Set Bedtimes Associated with Improved Sleep and Daytime Functioning in Adolescents. Sleep.

[25] Wolniczak I., Cáceres-DelAguila J.A., Palma-Ardiles G., Arroyo K.J., Solís-Visscher R., Paredes-Yauri S., Mego-Aquije K., Bernabe-Ortiz A. (2013). Association between Facebook Dependence and Poor Sleep Quality: A Study in a Sample of Undergraduate Students in Peru. PLoS ONE.

[26] Li S., Zhu S., Jin X., Yan C., Wu S., Jiang F., Shen X. (2010). Risk factors associated with short sleep duration among Chinese school-aged children. Sleep Med..

[27] Moretta T., Buodo G. (2018). Autonomic stress reactivity and craving in individuals with problematic Internet use. PLoS ONE.

[28] Reed P., Romano M., Re F., Roaro A., Osborne L.A., Viganò C., Truzoli R. (2017). Differential physiological changes following internet exposure in higher and lower problematic internet users. PLoS ONE.

[29] Balconi M., Campanella S., Finocchiaro R. (2017). Web addiction in the brain: Cortical oscillations, autonomic activity, and behavioral measures. J. Behav. Addict..

[30] Malo-Cerrato S., Martín-Perpiñá M.-D.-L.-M., Viñas-Poch F., Martín-Perpiñáis M.-D.-L.-M., Viñas-Pochis F. (2018). Excessive use of social networks: Psychosocial profile of Spanish adolescents. Comunication.

[31] Al-Dubai S., Ganasegeran K., Al-Shagga M.A.M., Yadav H., Arokiasamy J.T. (2013). Adverse Health Effects and Unhealthy Behaviors among Medical Students Using Facebook. Sci. World J..

[32] Wassef B., Kohansieh M., Makaryus A.N. (2017). Effects of energy drinks on the cardiovascular system. World J. Cardiol..

[33] Kozik T.M., Shah S., Bhattacharyya M., Franklin T.T., Connolly T.F., Chien W., Charos G.S., Pelter M.M. (2016). Cardiovascular responses to energy drinks in a healthy population: The C-energy study. Am. J. Emerg. Med..

[34] Narain A., Kwok C.S., Mamas M.A. (2016). Soft drinks and sweetened beverages and the risk of cardiovascular disease and mortality: A systematic reviw and meta-analysis. Int. J. Clin. Pract..

[35] Haslam D., McKeown N.M., Herman M.A., Lichtenstein A.H., Dashti H.S. (2018). Interactions between Genetics and Sugar-Sweetened Beverage Consumption on Health Outcomes: A Review of Gene–Diet Interaction Studies. Front. Endocrinol..

[36] Mangi A.M., Rehman H., Rafique M., Illovsky M. (2017). Energy Drinks and the Risk of Cardiovascular Disease: A Review of Current Literature. Cureus.

[37] United States Department of Health and Human Services (2014). The Health Consequences of Smoking—50 Years of Progress: A Report of the Surgeon General.

[38] Makadia L.D., Roper P.J., Andrews J.O., Tingen M.S. (2017). Tobacco Use and Smoke Exposure in Children: New Trends, Harm, and Strategies to Improve Health Outcomes. Curr. Allergy Asthma Rep..

[39] Peterson L.A., Hecht S.S. (2017). Tobacco, e-cigarettes, and child health. Curr. Opin. Pediatr..

